# Study protocol of a randomized controlled trial of fistula vs. graft arteriovenous vascular access in older adults with end-stage kidney disease on hemodialysis: the AV access trial

**DOI:** 10.1186/s12882-023-03086-5

**Published:** 2023-02-24

**Authors:** Mariana Murea, Ali I. Gardezi, Mathew P. Goldman, Caitlin W. Hicks, Timmy Lee, John P. Middleton, Roman Shingarev, Tushar J. Vachharajani, Karen Woo, Lama M. Abdelnour, Kyla M. Bennett, Duvuru Geetha, Lee Kirksey, Kevin W Southerland, Carlton J. Young, William M. Brown, Judy Bahnson, Haiying Chen, Michael Allon

**Affiliations:** 1 Department of Internal Medicine, Section on Nephrology, Wake Forest School of Medicine, Winston-Salem, NC USA; 2 Division of Nephrology, Department of Medicine, University of Wisconsin School of Medicine and Public Health, Madison, WI USA; 3 Department of Vascular and Endovascular Surgery, Wake Forest School of Medicine, Winston- Salem, NC USA; 4 Department of Surgery, Division of Vascular Surgery and Endovascular Therapy, Johns Hopkins University School of Medicine, 600 N Wolfe St, Halsted 668, Baltimore, MD USA; 5 Division of Nephrology, Department of Medicine, University of Alabama at Birmingham, Birmingham, AL USA; 6 Division of Nephrology, Birmingham Veterans Affairs Medical Center, Birmingham, AL USA; 7 Division of Nephrology, Department of Medicine, Duke University Medical Center, Durham, NC USA; 8 Department of Kidney Medicine, Cleveland Clinic, Cleveland, OH USA; 9 Department of Kidney Medicine, Glickman Urological & Kidney Institute, Cleveland Clinic Lerner College of Medicine, Cleveland Clinic, Cleveland, OH USA; 10 Division of Vascular Surgery and Endovascular Therapy, Department of Surgery, David Geffen School of Medicine, University of California, Los Angeles, Los Angeles, CA USA; 11 Department of Medicine, Division of Nephrology, David Geffen School of Medicine, University of California, Los Angeles, Los Angeles, CA USA; 12 Division of Vascular Surgery, Department of Surgery, University of Wisconsin School of Medicine and Public Health, Madison, WI USA; 13 Division of Nephrology, Department of Medicine, School of Medicine, Johns Hopkins University, Baltimore, MD USA; 14 Department of Vascular Surgery, Cleveland Clinic, Cleveland, OH USA; 15 Division of Vascular & Endovascular Surgery, Department of Surgery, Duke University Medical Center, Durham, NC USA; 16 Department of Surgery, University of Alabama at Birmingham, Birmingham, AL USA; 17 Department of Biostatistics and Data Science, Division of Public Health Sciences, Wake Forest School of Medicine, Winston-Salem, NC USA; 18 Department of Internal Medicine, Section on Nephrology, Wake Forest School of Medicine, Winston-Salem, NC USA; 19 Division of Nephrology, Department of Medicine, University of Alabama at Birmingham, Birmingham, AL USA

**Keywords:** Arteriovenous access, Fistula, Graft, Hemodialysis, Older adults

## Abstract

**Background:**

Treatment of end-stage kidney disease (ESKD) with hemodialysis requires surgical creation of an arteriovenous (AV) vascular access—fistula (AVF) or graft (AVG)—to avoid (or limit) the use of a central venous catheter (CVC). AVFs have long been considered the first-line vascular access option, with AVGs as second best. Recent studies have suggested that, in older adults, AVGs may be a better strategy than AVFs. Lacking evidence from well-powered randomized clinical trials, integration of these results into clinical decision making is challenging. The main objective of the AV Access Study is to compare, between the two types of AV access, clinical outcomes that are important to patients, physicians, and policy makers.

**Methods:**

This is a prospective, multicenter, randomized controlled trial in adults ≥ 60 years old receiving chronic hemodialysis via a CVC. Eligible participants must have co-existing cardiovascular disease, peripheral arterial disease, and/or diabetes mellitus; and vascular anatomy suitable for placement of either type of AV access. Participants are randomized, in a 1:1 ratio, to a strategy of AVG or AVF creation. An estimated 262 participants will be recruited across 7 healthcare systems, with average follow-up of 2 years. Questionnaires will be administered at baseline and semi-annually. The primary outcome is the rate of CVC-free days per 100 patient-days. The primary safety outcome is the cumulative incidence of vascular access (CVC or AV access)-related severe infections—defined as access infections that lead to hospitalization or death. Secondary outcomes include access-related healthcare costs and patients’ experiences with vascular access care between the two treatment groups.

**Discussion:**

In the absence of studies using robust and unbiased research methodology to address vascular access care for hemodialysis patients, clinical decisions are limited to inferences from observational studies. The goal of the AV Access Study is to generate evidence to optimize vascular access care, based on objective, age-specific criteria, while incorporating goals of care and patient preference for vascular access type in clinical decision-making.

**Trial registration:**

: This study is being conducted in accordance with the tenets of the Helsinki Declaration, and has been approved by the central institutional review board (IRB) of Wake Forest University Health Sciences (approval number: 00069593) and local IRB of each participating clinical center; and was registered on Nov 27, 2020, at ClinicalTrials.gov (NCT04646226).

**Supplementary information:**

The online version contains supplementary material available at 10.1186/s12882-023-03086-5.

## Background

There are currently about 600,000 U.S patients with end-stage kidney disease (ESKD) who are on chronic dialysis, and each year an additional 110,000 patients initiate dialysis [1]. About 90% of these patients receive hemodialysis (HD), and each individual is dependent on a vascular access as their “lifeline”, delivering blood to the extracorporeal circuit and dialysis machine [2]. Of the three types of vascular access for HD—arteriovenous (AV) fistula (AVF), AV graft (AVG), and central venous catheter (CVC)—CVCs are the least preferred choice, due to their association with frequent episodes of bacteremia and central vein stenosis [3–9]. Ideally, all patients with advanced chronic kidney disease would undergo timely pre-ESKD placement of an AVF or AVG, such that it would be ready for use when they initiate HD, and avoid CVC dependence. Unfortunately, approximately 82% of U.S. patients initiate HD with a CVC, either because they have not undergone pre-ESKD access surgery or because the access is not ready for use at the time of HD initiation [1]. This subset of patients remains CVC-dependent until a permanent access (AVF or AVG) can be placed and becomes suitable for cannulation. Moreover, if the initial AVF or AVG is abandoned, the patient again becomes CVC-dependent until a second AV access is placed and ready to use.

Until recently, the consensus guidelines on vascular access strongly preferred AVFs over AVGs, as in some populations AVFs have a longer secondary patency for dialysis and require less frequent surgical or percutaneous interventions to maintain their patency. On the other hand, a higher proportion of AVFs than AVGs are abandoned prior to their successful use, resulting in prolonged CVC-dependence. The superiority of AVFs over AVGs has been questioned, in particular for older (≥ 60 years) adults, who currently account for 60% of patients initiating chronic HD for treatment of ESKD [1]. Contemporary observational studies comprised of large cohorts of older adults have suggested that AVGs may confer similar or better patient outcomes than AVFs [10–12]. In an intention to treat analysis, AVGs had a higher secondary patency rate than AVFs in the first 18 months of access creation [13, 14]; shorter time to access cannulation; and lower rates of adjuvant procedures than AVFs, conferring faster transition to CVC-free HD [6, 15–17]. A meta-analysis of 13 studies concluded that older patients have a 50–65% higher risk of primary AVF failure and 80% higher risk of secondary AVF failure compared with younger patients [18].

### Rationale for the AV access study

Given the limitations of observational studies, there is a fundamental medical uncertainty about whether AVFs are truly superior to AVGs in older patients on HD. Given clinical outcome equipoise, there is a need for a definitive randomized controlled trial (RCT) to assess the relative merits of AVFs and AVGs in this subset of patients. A pilot RCT of AVG vs. AVF placement in older adults on maintenance HD with a CVC showed that enrollment and randomization to one of the two AV access surgeries is feasible [19, 20]. The AV Access Study RCT was designed as a multicenter trial to conclusively compare the effectiveness and safety of AVG vs. AVF in older adults receiving maintenance HD via CVC without a functional AV access. The overarching hypothesis is that AVGs will confer a higher rate of CVC-free days, fewer adjuvant procedures on the AV access, lower healthcare costs, and superior patient-reported outcomes compared with AVFs.

## Objectives

The *primary objective* of the AV Access study is to compare the relative effectiveness of AVFs and AVGs in maximizing CVC-free days.

### Rationale for the selection of CVC-free days as the metric of AV access intervention effectiveness

In clinical practice, the decision to establish an AV access revolves around the goal of removing the CVC in order to decrease patients’ risk of developing central venous stenosis and/or sepsis associated with use of CVCs. Between AVGs and AVFs, observational data suggest an imbalance in the rate of access outcomes that render patients CVC-free [9]. Generally, AVGs offer shorter time to maturation and successful cannulation—but might have shorter functional patency after successful use for HD. By contrast, AVFs have higher rates of primary failure; those that mature often require more interventions and longer times to maturation and successful cannulation—but might have longer functional span [9]. Once successful use for HD is established, AVFs require less frequent interventions than AVGs to maintain patency for HD. Therefore, AV access effectiveness in terms of CVC-free days encompasses pivotal, immediate and long-term, access events: rate of access maturation, time to access maturation and successful cannulation, partial or complete thrombosis, cannulation-precluding infection, and functional patency.

*Other objectives* of this study include comparing healthcare costs and patient-reported experiences between the two types of AV access approach, listed in Table 1.

**Table 1:** Study objectives, outcomes and measures

Objectives	Construct	Specific Measure	Source	Timing
	Primary Effectiveness Outcome			
Compare AV Access intervention effectiveness	CVC-free days	Rate of CVC-free days per 100 patient-days	Chart review	M0-End
	**Safety outcome**			
Compare access-related safety events	Rate of vascular access (CVC or AV access)-related severe infections	Hospitalizations or death due to AV access-related infections Hospitalizations or death due CVC access-related infections	Chart review	M0-End
	**Secondary Outcomes of Healthcare Costs**			
Compare vascular access-related healthcare costs	Healthcare costs, from the insurer perspective	Costs associated with vascular access care (index AV access, new AV access, and/or CVC)	Chart review	M0-End
	**Tertiary Outcomes of Patient-Reported Experiences**			
Characterize patient-reported outcomes	Satisfaction with AV access	Short-Form Vascular Access Questionnaire	Patient	M0, M6, M12, M18 & M24
	Health-related quality of life	EuroQol 5-dimension 3-level	Patient	M0, M6, M12, M18 & M24
	Regret with AV access intervention	Decision Regret Scale	Patient	M6, M12 M18 & M24
	Preferences	Attitude Scale (Tradeoffs Present/Future Health)	Patient	M0, M6, M12 M18 & M24
	Concordance between access approach and patient-reported goals of care	SUPPORT questionnaire	Patient	M0, M6, M12, M18 & M24
	**Other Outcomes**			
Compare vascular access-related outcomes	AV access primary failure	Rate of AV access maturation failure	Chart review	M0-End
	Time to AV access cannulation	Successful AV access cannulation	Chart review	M0-End
	AV access patency	Duration of AV access patency (primary, assisted, cumulative)	Chart review	M0-End
	CVC-related infections	Incidence rate of CVC-related infections per 100 patient-days	Chart review	M0-End
	AV access infections	Incidence rate AV access infections requiring or not requiring hospitalization, per 100 patient-days	Chart review	M0-End
	Adjuvant procedures	Endovascular or surgical procedures on AV access	Chart review	M0-End
	AV access infection rate	Local or systemic infections related to index AV access infection (e.g., cellulitis)	Chart review	M0-End
	CVC-related infection rate	Local or systemic infections related to CVC	Chart review	M0-End
	Hospitalization rate	Date and cause of hospitalization	Chart review	M0-End
	Survival	Date and cause of death	Chart review	M0-End
Evaluate relationships between preoperative physical function and index AV access outcomes	Preoperative frailty and index AV access outcomes	Grip Strength Chair stand test Pepper Assessment Tool for Disability Clinical Frailty Scale Index AV access primary failure Time to successful cannulation of index AV access Rate of adjuvant procedures on index AV access	Patient, Physician & Chart review	M0-End
M denotes month; M0, baseline; M6, month 6 etc.				

## Methods

### Study design

**Fig. 1:**
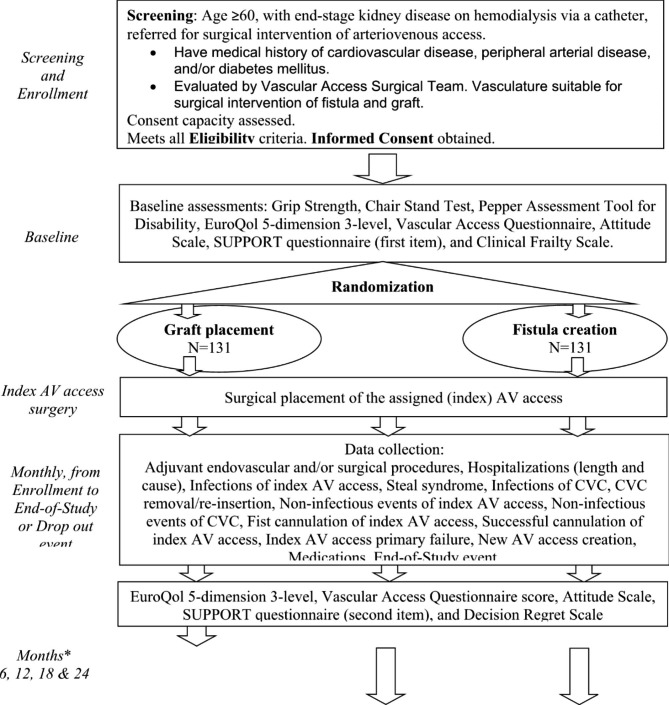
**Study Design Flow Diagram** *Assessment time points for AV patient-reported outcomes will be set from the date of index AV access surgery

This is a national, multicenter, individually-randomized, parallel-group controlled trial which targets enrollment of 262 elderly patients with ESKD with one or more coexisting medical conditions that puts the patient at higher risk of AVF maturation failure, i.e., cardiovascular disease, peripheral arterial disease and/or diabetes mellitus. Participants are randomized to surgical AVF creation vs. surgical AVG placement (Fig. 1).

The populated SPIRIT checklist for this study is provided as **Additional file 1.**

## Patient population

The study population is drawn from older adults, age 60 years and older, with ESKD receiving chronic HD via a CVC, who have been referred for AV access placement by their treating providers.

## Study setting

The study is conducted at seven national healthcare systems (**Additional file 2**). Enrollment and/or study-specific assessments take place at outpatient vascular access surgery clinics, outpatient nephrology clinics, outpatient or inpatient dialysis units, and inpatient nephrology or surgical services. All participating centers have established, large-volume nephrology and surgical practices that provide vascular access care for patients with ESKD.

## Eligibility criteria

These are listed in Table 2. Medical suitability for AV access surgery will be determined according to regional standard care for each patient. Anatomic and surgical suitability for AVF creation or AVG placement will be determined by the vascular access surgeon, according to usual care.

**Table 2:** Eligibility criteria

Inclusion criteria
1. Are of age 60 years or older
2. Have ESKD
3. Receive in-center HD as treatment for ESKD
4. Have CVC as the vascular access used for HD at the time of referral for AV access creation
5. Were referred by patient’s treating medical providers for placement of AV access
6. Have at least one of the following comorbid conditions: cardiovascular disease, peripheral vascular disease, and/or diabetes mellitus
7. Receive care for ESKD at medical facilities whereby the research team will have access to medical information (inpatient medical chart, outpatient medical chart, dialysis medical chart, outpatient interventional nephrology or interventional radiology procedures) during the study
8. Were evaluated by vascular access surgery and have native vasculature deemed preoperatively to be suitable for surgical creation of either type of AV access (AVF or AVG) in an upper extremity in the opinion of the surgeon
9. Are scheduled or will be scheduled for AV access surgery in an upper extremity, based on the plans developed by treating medical providers
10. Provide informed consent for study participation (for candidates with adequate consent capacity based on Consent Capacity Assessment tool) or obtain informed consent for study participation from a Legally Authorized Representative (for candidates unable to consent based on Decision-Making Capacity Assessment tool)
**Exclusion Criteria**
1. Receive home HD as treatment for ESKD
2. Anticipate kidney transplant within 6 months
3. Anticipate conversion to peritoneal dialysis within 6 months
4. Anticipate conversion to home HD within 6 months
5. Participate in another medical study, which, in the opinion of the site principal investigator, conflicts with this study
6. Have AVF creation planned by means other than surgical intervention (e.g. AVF creation is planned through endovascular surgery)
7. A condition in which, in the opinion of the site PI renders the patient not a good candidate for study participation.
AV, arteriovenous; AVG, AV graft; AVF, AV fistula; CVC, central venous catheter; ESKD, end-stage kidney disease; HD, hemodialysis.

## Recruitment

The approach to participant recruitment consists of two steps, prescreening and screening. At prescreening, clinic appointments are reviewed weekly by the study coordinators at each clinical center. For each patient ≥ 60 years old, scheduled for an evaluation of AV access creation, ESKD status, vascular access and comorbidities are appraised through review of electronic medical records. Those who pass prescreening are considered potential study candidates who will be screened at the time of their clinical appointment with and evaluation by the vascular access surgery. Patients who, in the opinion of the surgical team, have vasculature suitable for AVF creation and AVG placement in the upper extremity, will be approached for study participation. Before informed consent is obtained, the protocol requires the assessment of consent capacity for all study candidates using the using the Decision-Making Capacity Assessment Tool, enclosed in **Additional file 3**. The informed consent from the eligible candidate is obtained in a face-to-face interview with the patient or a Legally Authorized Representative for those without consent capacity.

## Randomization

After informed consent is obtained, a member of the study team at each clinical center enacts the randomization through the centralized REDCap platform (Fig. 1). The randomization system is based on a block-permutation scheme with variable (two to four) block sizes, and assigns participants in a 1:1 ratio to AVG or AVF placement, stratified by (a) clinical center and (b) history of previous AV access surgery.

## Blinding

Due to the visible nature of the intervention, blinding of the treating providers and investigators is not possible. Although the study intervention is unmasked, the clinical outcomes collected in the study are informed by medical diagnoses judged by the treating providers, independent of the study.

## Interventions

The study intervention is surgical placement of an AV access in an upper extremity (referred to as index AV access), either an AVF or an AVG, according to randomized allocation. Both types of AV access surgery are considered standard care. The specific vasculature used for index AVF or index AVG is at the discretion of the treating surgical team. The sites will use Food and Drug Administration (FDA)-approved grafts, with most common material being expandable polytetrafluoroethylene (ePTFE) grafts. Scheduling of the index AV access surgery date will occur according to local practices.

### Adherence to allocated intervention of index AV access surgery

After informed consent is obtained, the randomization assignment (i.e., type of index AV access to be surgically created) will be relayed to the vascular surgery team through verbal and electronic mail communication. Surgical intervention adherence to the assigned, index AV access is monitored during the study. All events of index AV access surgery postponement, missed surgery, or cancellation, with or without rescheduling, will be recorded along with the reason for such events.

### New AV Access surgery after index AV access surgery

If, over the course of follow-up, the index AV access is abandoned after its creation, the plan for and choice of a subsequent, new AV access will be at the discretion of the local medical team. Participants who undergo surgery for creation of a new AV access will continue to be followed until an end-of-study event. New AV access surgery will be subject to data collection similar to that of the index AV access surgery. AV access cannulation and complications during follow-up will identify whether it corresponded to the index AV access or a new AV access.

## Outcomes and measurements

All the outcomes, summarized in Table 1, are measured from the date of randomization to end-of-study event (i.e., drop out event or end-of-study date).

### Primary outcome

The primary outcome will compare the rate of CVC-free days following index AV access surgical creation. The total duration of CVC-free days will be determined for each patient and calculated for the whole cohort per 100 patient-days of study follow-up. In patients who undergo placement of a second AV access after abandonment of the index access, the total CVC-free days may include two or more discrete time segments of CVC independence interposed between periods of CVC dependence.

### Safety outcome

Safety between AVF and AVG strategy will be compared as aggregate rates of vascular access-related (CVC or AV access) infections that lead to hospitalization or death.

### Secondary, tertiary and other outcomes

The secondary outcome will compare vascular access-related healthcare costs; rates of AV access maturation; rates of AV access adjuvant procedures; rates of infectious and non-infectious vascular access (CVC or AV access) complications; and deaths. Event definitions for AV access outcomes and CVC outcomes, and cause of death categorization are listed in **Additional file 4**. Standard costs attributed to vascular access (index AV access, CVC, new AV access) adjuvant interventions and complications will be used to calculate cumulative access-related costs between the two groups (**Additional file 5**).

Tertiary outcomes consist of patient-reported outcomes. Patients’ satisfaction with their dialysis vascular access will be elicited with the Vascular Access Questionnaire [21] and the Decision Regret Scale [22]. Health-related quality of life will be assessed with the EuroQol 5-dimension 3-level [23]. Patient’s preferences between quality/quantity of life and future/present health will be assessed with the Attitude Scale [24] and SUPPORT questionnaire [25]. These instruments are described in **Additional file 6**.

Exploratory outcomes will evaluate for relationships between preoperative frailty measures and the rate of index AV access primary failure, time to successful AV access cannulation, and the rate of adjuvant procedures on index AV access.

## Participant timeline

Recruitment is projected over 2.5 years. We target an average follow-up period of 2 years from the date of randomization.

## Data collection

The schedule of assessments is summarized in Table 3. Participant’s baseline physical function will be assessed with objective tests (i.e., Grip Strength [26] and Chair stand [27]) and subjective instruments (i.e., Pepper Assessment Tool for Disability [28] and Clinical Frailty Scale [29]). Data will be collected prospectively through monthly review of electronic medical records for events documented at outpatient dialysis units, outpatient clinics, outpatient or inpatient interventional nephrology/interventional nephrology, and inpatient charts. We will record receipt of allocated index AV access (date of surgery, type of AV access surgery performed [AVF or AVG] and anatomical description, and attending surgeon’s expertise [number of new AV access surgeries performed in the 12 months prior to study start-up]). Medical events of interest, listed in **Additional file 7**, will be recorded with the diagnoses deemed by the treating medical team. Questionnaires will be administered pre-randomization and during follow-up at months 6, 12, 18 and 24 via telephone interviews. Time points for questionnaires administration are calculated from the date of randomization. The type(s) of vascular access a patient has at the time of questionnaire administration will be documented as either CVC, index AV access, and/or new AV access. At each clinical center, study coordinators responsible for questionnaire administration and data collection have received in-depth training in all study-related operational procedures, data collection, and data entry. Events of major or non-major protocol deviation and drop-out will be monitored and recorded (**Additional file 8**).

**Table 3:** Schedule of Evaluations

Assessment	Pre- & Screening	Baseline at randomization	Study Surgery	Schedule in Months			
				M6	M12	M18	M24
Medical history*	X	X					
Duplex ultrasound of vessels, pre-op^£^	X						
Consent Capacity Assessment		X					
Informed consent		X					
Demographics*	X						
Living situation		X					
Highest scholastic education achieved		X					
Medical Insurance*		X					
Nephrology care background*		X					
Medications*		X					
Blood laboratory data*		X (monthly)					
End-stage kidney disease history*	X						
Past AV access history*		X					
CVC history*		X					
Randomization group		X					
Surgeon expertise			X				
Index AV access description			X				
Antibiotic administered peri-op			X				
Grip Strength^†^		X					
Chair Stand test^†^		X					
Pepper Assessment Tool for Disability		X ^#^					
Clinical Frailty Scale^§^		X ^#^					
EuroQol 5-dimension 3-level^€^		X ^#^		X	X	X	X
Vascular Access Questionnaire score^€^		X ^#^		X	X	X	X
Attitude Scale (Tradeoffs)^€^		X ^#^		X	X	X	X
SUPPORT questionnaire^€^		X ^#^		X	X	X	X
Decision Regret Scale^€^				X	X	X	X
Duplex ultrasound of AV access				X (monthly)			
Hospitalizations (length and cause)		X (monthly)					
CVC complications^¶^		X (monthly)					
CVC removal / reinsertion		X (monthly)					
Adjuvant endovascular interventions^‡^				X (monthly)			
Adjuvant surgical interventions^‡^				X (monthly)			
AV access complications^¶^				X (monthly)			
Successful cannulation				X (monthly)			
AV access maturation failure				X (monthly)			
New AV access creation				X (monthly)			
Type of vascular access used for HD		X (monthly)					
End-of-study event^¥^		X (monthly)					
Serious Adverse Events		X (monthly)					

*Electronic medical records review

^£^Data collection when preoperative duplex ultrasound vascular mapping was performed

^†^Obtained in-person

^‡^Pertaining to AV access (index or new AV access) or CVC. ^§^Instrument will be completed by the treating physician. ^¶^Infectious and non-infectious complications and treatment

^¥^End-of-study event represents a drop out event (withdrawal of consent, withdrawal from the study, transition to peritoneal dialysis, transition to home HD, transfer of care outside participating health system network, kidney transplantation and successful discontinuation of HD, death) or end-of-study date

^€^Questionnaire administration between study coordinator and participant may take place in-person or via telephone

^#^Baseline questionnaires will be completed before index AV access surgery date or within 10 working days from the date of informed consent, whichever comes first

AV denotes arteriovenous; CVC, central venous catheter; HD, hemodialysis; M, month. Assessment time points for follow-up questionnaires will be set from the date of randomization

### Concurrent medical care

Participation in the study does not interfere with receipt of prescribed medications, medical devices or surgical procedures at any time point during the study. Diagnostic tests such as duplex ultrasound of the upper arm vasculature pertaining to an AV access will be part of standard care. Any adjuvant procedures required for the vascular access will be conducted as deemed necessary by the treating team. Findings from diagnostic tests (e.g., CT venogram, MR venogram) and procedural imaging (e.g., fistulogram) are logged in centralized REDCap study database.

## Statistical methods

### Sample size calculation and power considerations for primary outcome

The sample size estimate is based on the primary hypothesis that, by the end of the study, the patients in the AVG group will have more CVC-free days than patients in AVF group. Based on our pilot study [19, 20, 30], the average rate for CVC-free days was 15/100 patient-days among study patients who underwent AVF placement; similar or higher rate of CVC-free days was reported in other studies [31–33]. For this study, sample size calculations are based on two-sided tests with 5% type 1 error rate and a conservative estimate of 25% drop-out rate. Assuming a Poisson distribution and an average follow-up of two years, we will have > 90% power to detect a minimum of 5% points increase in CVC-free days in the graft group with 131 patients per group. We also considered situations where overdispersion is present for CVC-free days. With a total sample of 262 and 15/100 patient-days in the fistula group, we will have 80% power to detect an effect size of 25% for a 5% inflation of variance. The power will be greater if the rate of CVC-free days is 20/100 patient-days or higher in the fistula group.

### Analysis for primary outcome

The primary outcome is the rate of CVC-free days per 100 patient-days. The primary analysis will be performed under the intention-to-treat principle. The outcomes will be measured from the date of randomization to the date of an end-of-study event (see **Additional file 7**) or end-of-study date, whichever comes first. For each participant, the primary outcome will be the cumulative number of days that dialysis is delivered using AV access with the at-risk time as an offset. We will compare outcomes between the treatment groups using Poisson regression, modeling the number of CVC-free days as the dependent variable with a log link, the treatment assignment as the predictor, and the natural log of number of at-risk days as an offset. Covariate adjustment will include enrollment center and history of prior AV access. Analyses will take into account events of CVC re-use after CVC-free days. Deviance residuals and the overall deviance measure will be calculated to assess the overall goodness of fit of the model as part of model diagnostics. If we observe overdispersion, we will explore the negative binomial models. Sensitivity analyses will be performed in the as-treated and per-protocol populations.

As a supporting analysis, safety analysis will be conducted to examine severe access infections defined as infections requiring hospitalization or death caused by access infection. Rates of severe infections between AVG and AVF groups will be compared using Poisson regression models. If we observe overdispersion, we will explore the negative binomial models.

### Analysis for secondary outcomes

Access-related healthcare costs analysis will be based on the intention-to-treat principle. The costs will include index access placement (AVF or AVG), procedures required to promote study AV access maturation (angioplasty or surgical revision), procedures to maintain study AV access patency for HD after successful use (angioplasty, thrombectomy, or surgical revision), surgery to place a new vascular access (AVF or AVG) if the study AV access failed or was abandoned, procedures to promote maturation and maintain patency of all subsequent accesses, procedures of CVC exchange due to catheter dysfunction or infection, and reimbursement associated with access-related hospitalizations. Similar to our previous work, we will use the reimbursement fees established by the Center of Medicare and Medicaid Services, per each corresponding year of study, for access-related hospitalizations and inpatient or outpatient services and procedures [34]. Per-patient access cost will include the cost of all access-related procedures and hospitalizations divided by the total number of years of follow-up for that patient. As in standard economic analysis, costs will be discounted at 3% annually. Log transformation will be used to better approximate normality. A general linear model will be used to test the difference on the log scale between the fistula and graft groups. The estimates will be back transformed to represent median costs on the original scale. We will also use a generalized linear model with Gamma distribution and a log link to compare the mean access-related cost between the two groups.

Other secondary outcomes include rates of AV access maturation, rates of AV access adjuvant procedures, and rates of infectious and non-infectious vascular access (CVC or AV access) complications. Poisson regression models will be used to test the difference between AVF and AVG groups. If we observe overdispersion, we will explore the negative binomial models. Deaths will be analyzed using Cox regression models.

### Analysis for tertiary outcomes

Vascular Access Questionnaire scores will be obtained at 6, 12, 18 and 24 months during follow-up. We will use a linear mixed-effects model approach to compare the postoperative Vascular Access Questionnaire scores between AVF and AVG groups and to examine the longitudinal pattern in the Vascular Access Questionnaire scores over time within each treatment group. The model will include an indicator variable for treatment (AVF vs. AVG), time, and treatment by time interaction. Least square means for each group will be reported. We will test for treatment effect overall and at each time point using contrasts. Covariate adjustments will include baseline scores and the ones similar to the models used in the analysis for the primary outcome. For analyses of EQ-5D, Regret, and Tradeoffs, we will use a similar approach. For analysis of *goal concordant care regarding vascular access approach*, each participant will be classified as being concordance or not at each follow-up visit. A generalized estimating equation (GEE) model will be fit to compare the likelihood of being concordance between the AVG and AVF groups. Odds ratios (OR) averaged over time and at each time point will be reported.

### Analysis for exploratory outcomes

In these analyses we will assess the association between preoperative physical function and AV access primary failure. For these analyses, the primary predictor variable will be baseline physical function based on a composite score of grip strength, chair stand performance, clinical frailty score, and disability score. The outcome will be the incidence rate of fistula or graft primary failure. The cohort will be dichotomized at the median composite score. We will use the Cox proportional hazards model in each intervention group to explore the relationship between a baseline composite score of physical fitness and fistula or graft primary failure. Covariates included in the models will be similar to the primary analysis. The baseline composite score will additionally be modeled as a continuous variable and divided in tertiles. Receiver-operating curves will be used to compare the accuracy of the composite score that included all four markers of physical function and scores based on fewer physical function markers to a model that included the best clinical variables at predicting AV access primary failure. Areas under the curve will be compared with the method of DeLong and DeLong [35]. Similar analyses will be performed to separately test for associations between preoperative physical function and time to successful cannulation and incidence rate of adjuvant AV access procedures; and between each metric of physical function obtained at baseline and AV access primary failure.

### Missing data

Information collected during the study related to reasons that values are missing will be helpful in examining assumptions about missing data, e.g., whether data are missing completely at random (MCAR), missing at random (MAR), or missing not at random (MNAR). In general, all available data will be used in estimation and inference. Following the recommendations of the 2010 National Academy of Sciences report on the treatment of missing data in clinical trials, sensitivity analyses will be carried out to explore the effect of missing outcomes on inference for the primary outcomes. Multiple imputations will be used under MCAR and MAR assumptions. In the presence of informative censoring, shared parameter random effects models and/or pattern mixture models will be fit, and sensitivity analyses will be performed to check the robustness of study conclusions.

## Heterogeneity of treatment effects

We will test for heterogeneity of treatment effect as a follow-on exploratory analysis whenever a main treatment effect is detected, to determine whether the intervention has different relative benefits for different subgroups. ***Subgroup analyses***. We anticipate treatment effects will vary across different patient subpopulations defined by the following cofactors: age subcategories (< 80 vs. ≥ 80 years old at the time of enrollment) [11], sex [36–38], race [39, 40], place of residence at the time of enrollment (nursing home residence vs. other) [41], presence or absence of previous AV access creation [42, 43], and location of study AV access placement (forearm or arm) [15, 44−46]. Thus, subgroup analyses will be performed, and the tests will be based on the coefficients for the interaction terms between the intervention and the subpopulation cofactors.

## Safety evaluation and reporting of adverse events

Each participating investigator has primary responsibility for the safety of the individual participants under their care. Throughout the clinical trial, particular attention will be given to (serious) adverse events ((S)AEs). SAEs will be collected, documented and reported from enrollment to the end-of-study event or end-of-study date for each participant. All SAEs will have their relationship to study intervention, i.e., AVF or AVG placement, assessed by the principal investigator at each clinical center. The main principal investigators (MM and MA) are responsible for relaying all reportable SAEs to the central Institutional Review Board (IRB) of Wake Forest University Health Sciences and the National Institutes of Health (NIH)/National Institutes on Aging (NIA) Program Officer within 5 days of receipt of information event. An independent Data Safety Monitoring Board (DSMB) has been selected by the NIA Program Officer and is comprised of two clinicians and one biostatistician. The DSMB reviews the data generated throughout the study in a blinded manner, and may request unblinding for data review. The DSMB is informed of all reported SAEs by the NIA Program Officer. The entire clinical study might be discontinued upon unexpectedly high-frequency SAEs or an insufficient number of recruited patients.

## Data coordination and quality assurance

### Data management

All data obtained in the context of the clinical trial are subject to data protection. Data processing occurs on the legal basis of the patient’s informed consent to participate in this clinical study or the consent of his/her legal representative/authorized person or relative. Participants’ records and the data generated by the study will be confidential. The AV Access study data is captured and stored electronically via REDCap. The data extracted is de-identified and a unique subject number is used.

### Training

All data collectors have received training sessions that consisted of education on study protocol and induction to REDCap use and data entry. A Manual of Procedures for study-related activities was provided to all participating sites. Principal investigators and their co-investigators at each clinical center have met the following criteria: adequate time to conduct the study, adequate training and experience to conduct the study, ability to recruit enough participants to conduct the study, and provide evidence of proficiency in the tenets of Good Clinical Practice.

### Data monitoring and quality assessment

Every effort will be made to collect all data points in the study. The amount of missing data will be minimized by appropriate management of the trial, proper screening of patients, and training of participating investigators and study managers. Adherence to core components of the protocol is closely monitored and includes: (a) adherence to the assigned AV access surgery (i.e., creation of AVF in those randomized to AVF surgery; placement of AVG in those randomized to AVG surgery); (b) adherence to data collection through prospective, monthly review of electronic medical records regarding occurrence of events of interest; and (c) adherence regarding administration of questionnaires at specific time points.

## Dissemination policy

We will submit the findings of this study for peer-reviewed publication. Authorship eligibility will be determined using ICMJE guidelines [47]. Results will be presented at national and international conferences.

## Discussion

A large part of care of patients with ESKD is dedicated to the planning and creation of an AV access that is suitable for HD use, is least intrusive on the patient’s life, corresponds with the patient’s goals of care, and lowers healthcare costs. To navigate these imperatives, providers, policymakers, guideline working groups and patients have relied on retrospective, observational studies. Recent observational studies have suggested that, in older adults, AVGs may be a better vascular access strategy than AVFs, by conferring shorter duration of CVC use and fewer adjuvant procedures, yet not all studies evidenced these results [48]. A pervasive challenge is distinguishing whether the vascular access type *per se* directly affects clinical outcomes or whether there is a selection bias whereby the choice of vascular access approach and access development was a surrogate marker for the severity of comorbidities that themselves impact clinical outcomes [49]. For example, the decision to place an AVF in a patient may reflect a healthier clinical status in ways that are not captured even with sophisticated statistical analyses (e.g., perceived better prognosis, less severe comorbidities) [50, 51].

The AV Access RCT was designed to address weaknesses of prior research—i.e., lack of unbiased patient sampling—and render the two AV access groups comparable for both known and unknown baseline confounders. Because of this comparability, the effect estimates obtained in this study will more reliably estimate effects of exposure (i.e., type of surgical AV access) on outcomes unlikely to be explained by other factors, such as confounding or reverse association. Our study will quantify and compare AV access effectiveness in terms of net CVC-free days achieved after vascular surgery evaluation for AV access creation. Vascular access-related infectious complications will be captured and compared. Recent studies indicated that 2.3% of all deaths in patients on HD are access-related [51] and did not account for differences in patient survival by type of vascular access [52]. Large cohort-base data showed that, relative to AVG complications, lower rates of AVF infections are counterbalanced by the higher rate of CVC-related bloodstream infections incurred before AVF maturation [53]. Differences in healthcare costs are also anticipated [34], with AVG group incurring lower short-term costs (due to fewer adjuvant interventions and shorter time to cannulation) [20] but potential higher long-term costs (related to shorter patency span or higher rate of infectious complications) compared with the AVF group. Therefore, this study will comprehensively describe vascular access-related clinical outcomes to characterize the ‘balance’ of trade-off between CVC dependence, AV access failure and access complications between the two surgical AV access types.

To date, vascular access literature has been lacking on the impact of the two types of AV access, AVF vs. AVG, on patients’ health-related quality of life. This clinical trial will address this knowledge gap by collecting participants’ reports on vascular access satisfaction, decision regret, trade-offs, and concordance with goals of care. These data will broaden our understanding of patients’ experiences while navigating vascular access care and provide important information to clinicians when discussing vascular access choices with older adults.

Our study also explores novel predictors of AV access maturation. In current practice, the preoperative evaluation is centered on cardiovascular risk assessment and largely ignores physical function, disability, and their potential effect on access outcomes. Research outside the vascular access field showed that measures of physical performance are strongly associated with postoperative morbidity [54–56], hospitalization rate, and survival in older adults overall and those with ESKD [57–59]. In a few studies, pre- and postoperative handgrip exercises increased the diameter of the forearm veins, suggesting that this intervention could accelerate AV access maturation [60–62]. However, how standardized measures of physical performance are associated with AV access outcomes has not been examined. Our study will be first to assess whether there is an association between preoperative physical fitness and the rate of AV access maturation by integrating tools of objective (i.e., grip strength and chair stand test) and subjective (i.e., Pepper Assessment Tool for Disability and Clinical Frailty Scale) physical function assessment. The relationship between simple, bedside physical function studies and AV access maturation will broaden physicians’ decision-making tools to tailor AV access interventions and lower the prevalence of unsuccessful access surgeries in older adults, and to help inform the design of future studies to tailor AV access interventions.

One important limitation of this RCT is that we have restricted eligibility to patients who have already started HD. After careful consideration, we elected not to include pre-ESKD patients undergoing AV access surgery for several reasons. First, as these patients are not CVC-dependent, the primary study endpoint (duration of CVC-independence) would be less meaningful. Second, in the absence of a CVC, catheter-related infections (the primary safety outcome) would not occur. Third, it is not possible to definitively ascertain whether an AVF is mature until the patient starts HD and cannulation is attempted. In clinical practice, although it is preferred that patients undergo AV access surgery within 6–9 months prior to the anticipated start date of dialysis, time to HD initiation is often difficult to ascertain [63]. Fourth, pre-ESKD patients with an AV access tend to undergo fewer AV access adjuvant procedures than their counterparts on HD due to the fact that they are seen less often by their nephrology providers and there is less impetus to re-intervene on and accelerate development of an existing AV access when HD had not been initiated [53].

A second limitation of this study is the potential bias regarding physicians’ decision on whether a certain type of AV access might be preferentially considered on an individual basis. Although the recent observational studies support equipoise in clinical outcomes between AVF and AVG in older CVC-dependent HD patients with comorbid conditions, not all providers recognize such equipoise. To prevent the occurrence of ascertainment bias, emphasis was placed during training on the expectation that nephrology and surgery providers should exercise unbiased determination of surgical suitability for AVF and AVG among study candidates. When a potential candidate is deemed ineligible for either AVF or AVG, the reason for particular AV access ineligibility is documented and discussed during monthly web-based meetings with all site investigators.

In summary, the AV Access trial will address three essential questions pertaining to vascular access care for older adults on HD: (1) Which AV access confers better access-related outcomes? (2) What costs are associated with each AV access strategy? and (3) What level of satisfaction do patients report as they navigate different paths of vascular access care? The impact of this trial will be far-reaching by delineating a comprehensive array of access type-specific outcomes and exploring novel factors with a role in the mechanism of access failure. Results could transform the paradigm of vascular access care based on objective research and integration of patient-reported values.

## Trial status

This clinical study is currently in the enrollment phase. Enrolment started in June 2022, and the estimated completion date is the end of 2026. The study protocol uses the current version 4.4, dated October 28, 2020. The change history is given in the “Ethics and legal considerations” section.

## Electronic supplementary material

Below is the link to the electronic supplementary material.

Supplementary Material 1

Supplementary Material 2

Supplementary Material 3

Supplementary Material 4

Supplementary Material 5

Supplementary Material 6

Supplementary Material 7

Supplementary Material 8

## Data Availability

Only the principal investigators and the statisticians will have access to the final data set. The data sets of this study will be available from the corresponding author on reasonable request, after publication.

## Abbreviations

AV: arteriovenous
AVF: arteriovenous fistula
AVG: arteriovenous graft
CVC: central venous catheter
DSMB: Data Safety Monitoring Board
ESKD: end-stage kidney disease
HD: hemodialysis
ICMJE: International Committee of Medical Journal Editors
IRB: Institutional Review Board
NIA: National Institutes on Aging
NIH: National Institutes of Health
SAE: serious adverse events
