# Supplementary material for: Study protocol of a randomized controlled trial of fistula vs. graft arteriovenous vascular access in older adults with end-stage kidney disease on hemodialysis: the AV access trial
Source: BMC Nephrol. 2023 Feb 24;24:43. doi: 10.1186/s12882-023-03086-5 (PMC9960188; doi:10.1186/s12882-023-03086-5)
Supplement: Supplementary file 2 — Supplementary Material 2 [file 12882_2023_3086_MOESM2_ESM.docx]

**Additional file 2. Participating clinical centers**

Cleveland Clinic Lerner College of Medicine (CCLCM) of Case Western Reserve University School of Medicine (CWRUSM)

Cleveland, Ohio

Duke University School of Medicine (DUSM)

Durham, North Carolina

Johns Hopkins University School of Medicine (JHUSM)

Baltimore, Maryland

University of Alabama at Birmingham (UAB)

Birmingham, Alabama

University of California, Los Angeles (UCLA)

Los Angeles, California

University of Wisconsin School of Medicine and Public Health (UWSMPH)

Madison, Wisconsin

Wake Forest University School of Medicine

Winston-Salem, North Carolina
