# Supplementary material for: Study protocol of a randomized controlled trial of fistula vs. graft arteriovenous vascular access in older adults with end-stage kidney disease on hemodialysis: the AV access trial
Source: BMC Nephrol. 2023 Feb 24;24:43. doi: 10.1186/s12882-023-03086-5 (PMC9960188; doi:10.1186/s12882-023-03086-5)
Supplement: Supplementary file 3 — Supplementary Material 3 [file 12882_2023_3086_MOESM3_ESM.docx]

### **Additional file 3. Decision Making Capacity Assessment Tool**

### Consent Capacity Assessment

Potential Participant Name: ________________________________ Date:________

Brief Protocol Title:________________________________________ IRB #________

**ASSESSMENT QUESTIONS**:

1. Does the individual understand he/she would be participating in research and that research is voluntary?

Yes

No

2. Does the individual understand what will happen to him/her if he/she decides to participate?

Yes

No

3. Does the individual know how long he/she will be in the research study?

Yes

No

4. Can the individual explain one or two risks associated with the research study?

Yes

No

5. Can the individual explain what he/she should do to stop being in this research study?

Yes

No

6. Does the individual know who to contact if he/she experiences problems or has questions about the study?

Yes

No

7. Can the individual explain what alternatives there are if he/she chooses not to participate?

Yes

No

**Judgment**

Based on the sample criteria and the person’s responses, do you believe s/he has the decision-making capacity to give informed consent for this study? ____YES ____NO

______________________________________________________________________

Signature of Evaluator Date
