# Supplementary material for: Study protocol of a randomized controlled trial of fistula vs. graft arteriovenous vascular access in older adults with end-stage kidney disease on hemodialysis: the AV access trial
Source: BMC Nephrol. 2023 Feb 24;24:43. doi: 10.1186/s12882-023-03086-5 (PMC9960188; doi:10.1186/s12882-023-03086-5)
Supplement: Supplementary file 4 — Supplementary Material 4 [file 12882_2023_3086_MOESM4_ESM.docx]

| **Additional file 4. Definitions for vascular access outcomes** (according to the recommendations of the Committee on Reporting Standards for Arteriovenous Accesses, Society for Vascular Surgery and American Association for Vascular Surgery (Shenoy et al, Clin J Am Soc Nephrol. 2018;13(3):490-494) (Beathard et al., Clin J Am Soc Nephrol. 2018;13(3):501-512). | |
| --- | --- |
| ***AV access outcome*** | ***Definition and Examples*** |
| AV access primary failure | Permanent failure of the fistula or graft before successful access cannulation for HD. This includes inadequate maturation, thrombosis, failure of first and subsequent cannulations, and other complications leading to nonfunctional or unusable fistula or graft  Primary maturation failure will be further sub-classified as immediate (within 72 hours of access creation), early (by the end of month 3 following access creation), or late failure (by the end of month 6 following access creation) |
| Successful cannulation of AV access | The arteriovenous access became the primary vascular access for hemodialysis (the fistula or graft access has been cannulated, CVC was removed, and the index AV access became the primary and sole vascular access for HD) |
| Unassisted access maturation | Criteria for fistula or graft suitable for HD (based on the above criteria) are met before any endovascular or secondary surgical procedure to facilitate maturation |
| Assisted access maturation | Successful cannulation of fistula or graft for HD after a procedure to facilitate maturation (e.g., angioplasty, stent placement, surgical revision, ligation of accessory veins) |
| Primary access patency | Intervention-free access survival defined as the time from fistula or graft creation to any intervention to establish or maintain patency |
| Secondary access patency | AV access survival defined as the time from intervention to establish or maintain AV access patency to permanent AV access failure or abandonment |
| Secondary access failure | Permanent failure after the fistula or graft was successfully used for HD with subsequent AV access abandonment |
| Noninfectious complications | Stenosis, thrombosis, hand ischemia/steal syndrome, aneurysm, pseudoaneurysm, infiltration, seroma, hematoma |
| Infectious complications | Fistula or graft cellulitis, abscess, bacteremia |
| Access procedures | Angioplasty, stent placement, surgical revision, ligation of accessory veins, superficialization of vein, (pseudo)aneurysm resection, ligation, graft explant, abscess evacuation, hematoma evacuation, seroma/fluid collection drainage |
| Other clinical outcomes | new AV access surgical creation, AV access–related hospitalization or death |
| ***CVC access outcome*** | ***Examples*** |
| Infectious complications | CVC exit site infection, tunnel infection, bacteremia |
| Non-infectious complications | CVC removal, CVC exchange over guidewire, CVC placement,  CVC malposition, mechanical dysfunction, catheter migration, venous thrombosis, pneumothorax, hemothorax |
