# Supplementary material for: Study protocol of a randomized controlled trial of fistula vs. graft arteriovenous vascular access in older adults with end-stage kidney disease on hemodialysis: the AV access trial
Source: BMC Nephrol. 2023 Feb 24;24:43. doi: 10.1186/s12882-023-03086-5 (PMC9960188; doi:10.1186/s12882-023-03086-5)
Supplement: Supplementary file 5 — Supplementary Material 5 [file 12882_2023_3086_MOESM5_ESM.docx]

**Additional file 5**

The objective of the economic evaluation is to compare the within-trial total vascular access-related (index AV access, CVC, new AV access) costs between the AVF and AVG groups. The total costs will be comprised of costs for vascular access-related procedures and hospitalizations.

**Cost for Vascular Access-Related Procedures**

The 2022 Current Procedural Terminology (CPT) codes will be assigned to the pre-specified vascular access-related procedures (Table S1). For some procedures multiple CPT codes may be assigned to represent all related professional services. The 2022 Medicare Physician Fee Schedule (MPFS) will be used to assign physician fees. Non-facility fees will be used to calculate costs for procedures performed in the outpatient settings. Facility fees will be used to calculate costs for procedures performed in the ED or inpatient settings.

**Cost for Vascular Access-Related Hospitalizations**

For each vascular access-related hospitalization, we will calculate the length of stay (LOS) and record the presence of any ICU stay and mechanical ventilation of >=96 hours. The 2022 Diagnosis Related Groups (DRG) codes will be assigned to the pre-specified discharge diagnosis of each vascular access-related hospitalization (Table S2). For each DRG code, we will divide the 2022 Medicare reimbursement by the median LOS published by CMS to estimate the cost per inpatient day. This daily reimbursement amount will be used to estimate the DRG-based cost for hospital care.

Another component for the comprehensive hospitalization cost is the physician fees for inpatient services that are not included in the DRG-based reimbursement. We will use the following CPT codes to calculate the costs for the day of admission, the day of discharge, and the days in between (Table S3).

| **Table S1. The pre-specified vascular access-related procedures** | | |
| --- | --- | --- |
| Vascular Access Type | Procedures | 2022 CPT Codes |
| CVC | | |
| Procedures | CVC insertion | 36010, 36011, 36012, 36800,  36578, 36580, 36581, 36582, 36583, 77001 |
|  | CVC removal | 36589,  36578, 36580, 36581, 36582, 36583, 77001 |
| AV access | | |
| Imaging | Ultrasound (Vascular Ultrasound, Duplex Ultrasound or Doppler Ultrasound) | 93990 |
|  | CT (or MRI) chest with contrast or CT (or MRI) Venogram of the chest | 71260, 71551 |
| Adjuvant percutaneous/endovascular procedures | Angiography | 36901, 36902, 36907 |
|  | Thrombectomy or Thrombolysis of AV access | 36904, 36905, 36906, 37187, 37188 |
|  | Stent placement | 36903, 37238, 37239 |
|  | Embolization of Accessory Vein(s) or Embolization of AV access | 36909 |
| Adjuvant surgical/open procedures | Superficialization of AV access | 36818, 36819, 36820, 36832 |
|  | open (surgical) procedure (a.k.a. blood clot removal) | 36831, 36833 |
|  | Evacuation of a hematoma | 35860, 35875, 35876 |
|  | Abscess draining or Abscess evacuation | 35860 |
|  | Access revision | 35236, 35266, 36832 |
|  | AV access ligation | 37607 |
|  | AV access removal/extraction/resection | 35903 |
|  | (Pseudo)Aneurysm repair or (Pseudo)Aneurysm resection or (Pseudo)Aneurysm plication or Aneurysmorrhaphy | 35011, 35013, 35045, 36832 |
|  | Accessory Vein(s) ligation or Accessory Veins closure or Accessory Veins resection | 36832 |
|  | Steal syndrome surgery | 36832, 36838 |

| **Table S2. The pre-specified discharge diagnosis** | 2022 DRG code |
| --- | --- |
| AV access surgical creation | 264 |
| AV access-related infectious complication with ICU stay and with MV | 870 |
| AV access-related infectious complication without ICU stay and with MV | 870 |
| AV access-related infectious complication with ICU stay and without MV | 871 |
| AV access-related infectious complication without ICU stay and without MV | 872 |
| AV access-related non-infectious complication with ICU stay and with MV | 314 |
| AV access-related non-infectious complication without ICU stay and with MV | 314 |
| AV access-related non-infectious complication with ICU stay and without MV | 315 |
| AV access-related non-infectious complication without ICU stay and without MV | 316 |
| CVC access-related infectious complication with ICU stay and with MV | 870 |
| CVC access-related infectious complication without ICU stay and with MV | 870 |
| CVC access-related infectious complication with ICU stay and without MV | 871 |
| CVC access-related infectious complication without ICU stay and without MV | 872 |
| CVC access-related non-infectious complication with ICU stay and with MV | 314 |
| CVC access-related non-infectious complication without ICU stay and with MV | 314 |
| CVC access-related non-infectious complication with ICU stay and without MV | 315 |
| CVC access-related non-infectious complication without ICU stay and without MV | 316 |

* MV: Mechanical Ventilation >=96 hours

| **Table S3. Physician fees for inpatient services** | | |
| --- | --- | --- |
| Inpatient services | 2022 CPT code | Description |
| Admission day | 99223 | Initial hospital inpatient care, typically 70 minutes per day |
| Discharge day | 99239 | Hospital discharge day management, more than 30 minutes |
| Other days | 99233 | Subsequent hospital inpatient care, typically 35 minutes per day |
