# Supplementary material for: Study protocol of a randomized controlled trial of fistula vs. graft arteriovenous vascular access in older adults with end-stage kidney disease on hemodialysis: the AV access trial
Source: BMC Nephrol. 2023 Feb 24;24:43. doi: 10.1186/s12882-023-03086-5 (PMC9960188; doi:10.1186/s12882-023-03086-5)
Supplement: Supplementary file 6 — Supplementary Material 6 [file 12882_2023_3086_MOESM6_ESM.docx]

**Additional file 7. Events of interest and categorization**

**End-of-study events**

End-of-study events are events that indicate a study finishing point with no further follow-up. They include drop out events and end-of-study date:

- Participant death
- Participant withdrew consent
- Participant withdrawn from the study by the PI
- Withdrawal of HD by the treating team
- Transfer of nephrology care outside participating health system network
- Kidney transplantation
- Transition to peritoneal dialysis
- Transition to home HD
- Lost to follow-up
- End-of-study date

**Key study events**

Key events are clinical events important in relation to study outcomes measures. These events will be collected from date of enrollment to an end-of-study event. These data will be extracted through thorough electronic medical chart review on a monthly basis. Key study events are summarized in include:

- Complications of index AV access
  - Hematoma
  - Seroma
  - Infection (cellulitis, abscess)
  - Bleeding (prolonged bleeding)
  - Thrombosis
  - Stenosis
  - Steal syndrome
- Complications related to CVC
  - Infection
  - Malfunction
- Hospitalizations
- Endovascular adjuvant procedures on AV access
- Surgical adjuvant procedures on AV access
- Endovascular or surgical procedures on CVC
- Successful AV access cannulation
- Date of CVC removal due to successful use of AV access
- Date of CVC reinsertion due to AV access malfunction
- New AV access placement
- Death

**Adjuvant Procedures**

Endovascular and surgical adjuvant procedures may occur during the study. All adjuvant procedures will be recorded with date, type of procedure, and reason for procedure.

*Endovascular adjuvant procedures involving the AV access include*:

- - Percutaneous thrombectomy
  - Percutaneous revision of anastomosis or dilation of vein/artery (e.g., angioplasty) with or without stent placement
  - Dilation (angioplasty) of central venous stenosis
  - Embolization or beads-based occlusion of tributaries or accessory veins

*Surgical adjuvant procedures involving the AV access include*:

- - Surgical thrombectomy
  - Dilation of central venous stenosis
  - Surgical revision of anastomosis or dilation of vein/artery (e.g., angioplasty) with or without stent placement
  - Ligation of tributaries or accessory veins
  - Superficialization of index AVF
  - Surgical explant of index AVG
  - Evacuation / Drainage of hematoma
  - Evacuation / Drainage of seroma
  - Evacuation / Drainage of abscess
  - Evacuation / Drainage of fluid collection
  - Second-stage planned procedure of index brachio-basilic AVF
  - Ligation of AVF or salvage by distal reconstruction and interval ligation) due to distal ischemia (steal syndrome)

| **Drop-outs and other events** | |
| --- | --- |
| **Drop out**^§^ | **Cause/Consequences** |
| Withdrawal of consent | Participant requested withdrawal from study |
| Withdrawal from the study^‡^ | Participant withdrawn from study by the Investigator |
| Transition to peritoneal dialysis | Dialysis therapy changes from HD to peritoneal dialysis |
| Transition to home HD | Dialysis therapy changes from in-center HD to home-based HD |
| Transfer of care outside participating health system network | Medical care transferred outside participating Clinical Centers Networks |
| Kidney transplantation with successful discontinuation of HD^¶^ | HD is discontinued |
| Death | Cause of death |
| **Other events** | **Cause/Consequences** |
| Cancellation of index AV access surgery* | Medically unstable  Change in goals of care for vascular access |
| Surgery for AV access did not take place before end-of-study event (and AV access surgery was not cancelled)** | Participant refuses AV access surgery  Intercurrent illness that lead to surgery postponement |
| Index AVF or index AVG is not usable, even after salvage procedures and several attempts | Primary or secondary AV access failure |
| Index AVF or index AVG is usable but complication rate is high | Steal syndrome  Infection and AV access explant  AV access ligation |
| Index AVF or index AVG is usable but needling is not tolerated by the patient | Participant refuses to have AV access cannulated |
| *Participants whose AV access surgery is permanently cancelled will be followed till drop out event (withdrawal of consent, withdrawal from the study, transition to peritoneal dialysis, transition to home HD, transfer of care, kidney transplantation and successful discontinuation of HD, death) or end-of-study date.  **Participants whose AV access surgery continued to remain postponed/unaccomplished will be followed till drop out event (withdrawal of consent, withdrawal from the study, transition to peritoneal dialysis, transition to home HD, transfer of care, kidney transplantation and successful discontinuation of HD, death) or end-of-study date.  ^§^Participant data is censored on the date of drop out.  ^‡^Reason for participant withdrawal by the PI is documented in related study form.  ¶ Participants who undergo kidney transplantation will be followed until HD treatments are discontinued. Those with unsuccessful kidney transplantation who continue to require HD will remain in the study. | |

| **Classification of causes of death** |
| --- |
| **Non-vascular access etiology** |
| 1. *Cardiovascular* |
| Acute coronary syndromes and related complications  Heart failure  Cardiac dysrhythmias  Valvular disorders  Myocardial disorders  Pericardial disorders  Complications of cardiac or vascular surgery  Complications of cardiac or vascular procedures  Cardiac perforation following hemodialysis catheter insertion  Cardiac arrest during placement of a dialysis catheter in radiology suite  Complications of peripheral vascular disease (examples: ischemic gut, ischemia of the extremities)  Complications of aortic dissection and aortic aneurysm  Complications of congenital heart disease |
| 2. *Cerebrovascular* |
| Acute stroke syndromes and complications (ischemic and/or hemorrhagic)  Subdural hemorrhage  Subarachnoid hemorrhage  Ruptured cerebral aneurysm  Other |
| 2. *Sudden death* |
| Sudden death without antecedent illness or procedure that could have contributed to the death. |
| 3. *Infectious* |
| Death related to COVID-19 infection  Bacteremia/sepsis from source other than hemodialysis vascular access.  Examples of sources of sepsis: pneumonia, urinary tract, infective endocarditis, intra-abdominal abscess, colitis, septic joint, soft tissue ulcer, etc. |
| 4. *Malignancy-related* |
| Complications of any malignancy leading to death. This includes metastatic disease leading to withdrawal of care or palliation. |
| 5. *Elective withdrawal from dialysis* |
| Elective discontinuation of dialysis therapy, in the absence of an acute, antecedent precipitant such as an acute medical illness. |
| 6. *Other* |
| Any other condition that did not fall into one of the 5 preceding categories: admission for failure to thrive with sudden death in the hospital and no clear precipitant, admission for bowel perforation and died in hospital from complications, motor vehicle accident, trauma, suicide, etc. |
| **Vascular access, related to index AV access** |
| Infectious, including index AV access related bacteremia, sepsis, endocarditis, or other terminal event caused by index AV access infection.  Non-infectious, caused by a non-infectious complication of index AV access. |
| **Vascular access, related to CVC** |
| Infectious, CVC-related bacteremia, CVC-related sepsis and infective endocarditis, CVC-related sepsis and osteomyelitis, or other terminal event caused by CVC infection.  Non-infectious, caused by a non-infectious complication of CVC. |
| **Vascular access, related to new AV access** |
| Infectious, including index AV access related bacteremia, sepsis, endocarditis, or other terminal event caused by index AV access infection.  Non-infectious, caused by a non-infectious complication of index AV access. |
