# Supplementary material for: Study protocol of a randomized controlled trial of fistula vs. graft arteriovenous vascular access in older adults with end-stage kidney disease on hemodialysis: the AV access trial
Source: BMC Nephrol. 2023 Feb 24;24:43. doi: 10.1186/s12882-023-03086-5 (PMC9960188; doi:10.1186/s12882-023-03086-5)
Supplement: Supplementary file 7 — Supplementary Material 7 [file 12882_2023_3086_MOESM7_ESM.docx]

| **Additional file 8**. **Protocol deviations** | |
| --- | --- |
| **Protocol Deviation*** | **Cause** |
| ***Major deviations*** | |
| Deviation from informed consent protocol | Consent capacity was not assessed |
| Receipt of an AV access that differs from the randomized intervention | Inadvertent occurrence  Intra-operative decision by surgery team |
| Deviations involving participant confidentiality | Breach in protection of participant confidentiality |
| ***Non-major deviations*** | |
| Failure to undergo surgery for AV access on the scheduled date | Participant did not present for surgery  Postponed surgery for AV access due to participant’s medical condition  Postponed surgery for AV access for other reasons (e.g., staffing issues related to COVID-19 pandemic) |
| Failure to collect data on questionnaires within a timeframe larger than ±14 days from a study time point | Participant refused  Unable to reach the participant  Unable to be provided by the participant (e.g., subject ill, intubated, hospitalized) |
| Failure to collect data on physical function assessment at baseline | Participant refused  Unable to stand/sit  Deemed unable to perform stand/sit test |
| Failure to review the medical chart prospectively, monthly | Inadvertent occurrence |
| *Participants whose AV access surgery is permanently cancelled will be followed till drop out event (withdrawal of consent, withdrawal from the study, transition to peritoneal dialysis, transition to home HD, transfer of care, kidney transplantation and successful discontinuation of HD, death) or end-of-study date. | |
