# Supplementary material for: Study protocol of a randomized controlled trial of fistula vs. graft arteriovenous vascular access in older adults with end-stage kidney disease on hemodialysis: the AV access trial
Source: BMC Nephrol. 2023 Feb 24;24:43. doi: 10.1186/s12882-023-03086-5 (PMC9960188; doi:10.1186/s12882-023-03086-5)
Supplement: Supplementary file 8 — Supplementary Material 8 [file 12882_2023_3086_MOESM8_ESM.docx]

**Additional file 6. Assessment instruments during the AV Access Trial**

**1. Grip Strength Protocol**

Hand grip strength is a commonly used measure of upper body skeletal muscle function and has been widely used as a general indicator of frailty with predictive validity for both mortality and functional limitation (see reference 26), cited with the manuscript main text). Other than possible temporary discomfort during the test itself, there are no known risks for the participant.

**
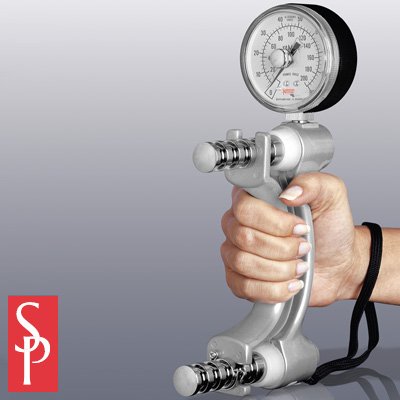
Required Equipment & Personnel**

The Jaymar Handheld Dynamometer is used to measure grip strength. The dynamometer is a precision instrument and its accuracy can be impaired by abuse. Never force the handles or subject the instrument to unnecessary impact.

**Exclusion Criteria**

If the participant reports current flare-up of pain in the wrist or hand, or has undergone fusion, arthroplasty, tendon repair, synovectomy, or other related surgery of the hand or wrist in the past 3 months, the affected side should not be tested, and the “Unable to test/exclusion” box should be marked for the trials on that hand. If the participant is unable to test or is excluded from testing both hands due to recent worsening of arthritis pain and/or surgery on hands/wrists within the past 3 months, mark “Unable to test/exclusion” on all three trials and move on to the next test.

Additional file 6 Figure 1: Measuring grip strength with Jaymar dynamometer

**Procedures**

This test should be done with the participant in a seated position. Determine whether the participant is right- or left-handed.


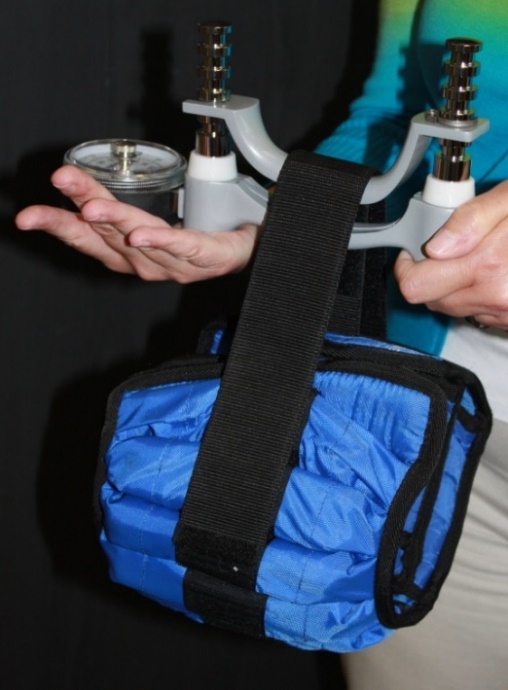
Set the dynamometer handgrip at **position two.** Adjust it for a smaller or larger hand when necessary. To adjust the handgrip handle, remove/pull out the handle clip located at the lower post (furthest from the gauge). Move the handle one position out (larger) or in (smaller) at both the bottom and top and re-secure/push in the handle clip. If the handle is not replaced in the correct position, the readings will not be accurate. Check that the arrow is set at ZERO.

The dynamometer is fairly heavy, so caution the participant when handing them the instrument. Allow one practice try to familiarize the participant with the feel of the instrument. Ensure that the bars are the proper distance apart for a comfortable grip. The participant’s arm should be resting on the table with the elbow bent.

Test both hands. Test the right hand first and then the left hand, with a total of three trials for each hand. Record each value to the nearest 2 kilograms, e.g. 40 (kg). If <10 kg, right justify and zero fill, e.g. 8 (kg) = 08. After each reading, reset the arrow to ZERO. Results of the three trials on each hand should be within 4 kg. If the trials differ by more than 4 kg, have the participant do a fourth trial on the hand(s) that differs by >4 kg.

Additional file 6 Figure 2: Equipment calibration

For each measurement, instruct the participant to squeeze as hard as they can. Allow 10 seconds between each measurement. Discontinue a measurement with anyone complaining of pain, then code as ‘unable’.

**Equipment Calibration**

The dynamometer calibration should be checked and logged on a monthly basis (see log on following page). Calibration should also be checked if the device is dropped or mishandled. This is done by **slowly** lifting the following weights strapped to the handle (see figure): 10 pounds, 20 pounds, and 30 pounds. Each weight should be lifted twice and the results recorded. The average dial reading for each weight should be within 4 pounds of the reference weight. If the dynamometer fails calibration, do not use it, and contact the CCC (Benjamin Bagwell 336-716-5777 or bbagwell@wakehealth.edu) immediately for a replacement.

**Hand Dynamometer Calibration Checks**

| **Date** | **10 pounds** | | **20 pounds** | | **30 pounds** | |
| --- | --- | --- | --- | --- | --- | --- |
|  | **Lift 1** | **Lift 2** | **Lift 1** | **Lift 2** | **Lift 1** | **Lift 2** |
|  |  |  |  |  |  |  |
|  |  |  |  |  |  |  |
|  |  |  |  |  |  |  |
|  |  |  |  |  |  |  |
|  |  |  |  |  |  |  |
|  |  |  |  |  |  |  |
|  |  |  |  |  |  |  |
|  |  |  |  |  |  |  |
|  |  |  |  |  |  |  |
|  |  |  |  |  |  |  |
|  |  |  |  |  |  |  |
|  |  |  |  |  |  |  |
|  |  |  |  |  |  |  |
|  |  |  |  |  |  |  |
|  |  |  |  |  |  |  |
|  |  |  |  |  |  |  |

Checks should be completed monthly plus any time the dynamometer is dropped or
mishandled. The average dial reading (2 readings at each weight) should be within 4 pounds
of the tested weight.

**2. Chair Stand Test Protocol**

Description:

The time taken for the participant to rise from sitting in a chair 5 times is measured. The test is completed without using hands on the chair or other tools to help the participant stand (see reference 27).

Equipment:

Standard height chair (43-45 cm, 17-18 inches) with a backrest

Stopwatch

Setup:

Patient is seated on a straight-backed chair and asked to stand up 5 times as quickly as possible with arms folded across his/her chest; time to complete 5 chair rises is recorded (cue to stop stopwatch is when patient is standing after fifth chair rise)

Administration:

- One trial is administered.
- A patient is instructed to sit with arms folded across their chest and with back against the chair. A patient with hemiplegia can have the impaired arm at his/her side or in a sling.
- If individuals are unable to complete the first sit to stand independently, without use of arms, the test is terminated.

Prompts:

- “We would like to evaluate your ability to sit up in a chair 5 times. Using a stop watch, we will record the time it took you to do this test. The score is the amount of time (to the nearest decimal in seconds) it takes a patient to transfer from a seated to a standing position and back to sitting five times.”

Or

- “I want you to stand up and sit down five times in a row, as quickly as you can, when I say ‘Go’. Be sure to stand up fully and try not to let your back touch the chair back between each repetition. Do not use the back of your legs against the chair.”
- Time starts when the tester says “Go.”
- Time stops when the patient’s body touches the chair following the fifth repetition.

Scoring:

Document the time in seconds (to the nearest decimal) required to complete the test.

If the patient cannot perform five stands to complete the test without use of arms, a score of 0 seconds should be documented. When possible within the medical record it is also recommended to note the reason, such “unable to perform five repetitions.” The tester can

**Points Performance**

0 Unable or ≥60 sec

1 ≥16.70– 59.99 sec

2 13.70–16.69 sec

3 11.20–13.68 sec

4 ≤11.19 sec

**Detailed Description of the Chair Stand Test**

In this test, participants are first instructed to fold their arms across their chest and to try to stand up one time from an armless chair placed against a wall. To perform this test you will need a stopwatch, the script, and a straight-backed chair with a hard seat. If this type of chair is not available, a chair with a softer seat or a chair with arms may be substituted.

If the participants are successful rising from the chair once, they are then asked to stand up and sit down 5 times as quickly as possible. Timing begins as soon as the command to stand is given and continues until the participants straighten at the end of the fifth stand. When learning to do this, it is useful for two or more people to time the test so that the times can be compared for precision.

For efficiency, it is valuable to have two chairs available so that the examiner can do the demonstration while the participants sit in the other chair and watch. If only one chair is available then the participants will have to get up to watch the demonstrations.

To ensure safety, the examiner should stand in front of the participants and be prepared to catch them if they fall forward. However, do not stand so close that the participants feel hemmed in and slow their pace during the chair stands.

For the first portion of the test, simply record whether the participants were able to rise from the chair without the use of their arms. If the participants are unsuccessful, the examiner should ask the participants to try to stand using their arms. Inability to complete the single chair stand with arms folded or being able to do it only with use of the arms ends the chair stand test.

For the second portion of the test (multiple chair stands), instruct the participants to stand up straight as quickly as they can five times without stopping in between. After standing up each time, the participants must sit down and then stand up again, keeping their arms folded across their chest. Emphasize the word “quickly” and perform the demonstration quickly to further reinforce this point. Count the stand number only after the participant has straightened up. Do not pace the test with your counting. If the participants do not stand up or sit down completely, stop the test and re-demonstrate.

Timing begins when the command to stand is given and continues until the participants straighten their body at the end of the fifth rise. During the test, count out loud as the participants rise up to five times. Do not coach or encourage the participants during the test. Watch the participants closely and stop the test if the participants are tired or short of breath during the repeated chair stands. The test should be stopped if the participants have to use their arms to rise at any time or if the participants have not completed the five chair rises after one minute. You should also stop the test at your discretion if, for any reason, you are concerned about the participant’s safety. If the participants stop before completing the five rises, you should ask them if they can continue. If the participants say yes, continue timing. If the participants say no, stop the test. If the participant is unable to perform all 5 tests, the score will automatically be a zero.

**3. The Pepper Assessment Tool for Disability (PAT-D) Questionnaire**

The PAT-D assesses the participant’s ability to complete an array of important day-to-day activities without difficulty and, for a subset of activities, without personal assistance (see reference 28). This information will allow us to determine whether the study interventions improve the ability to manage day-to-day activities. For this questionnaire, the interviewer is instructed to show the participant a response card, which will include the possible responses for all questions. This card should facilitate ascertainment of accurate responses and minimize the need to repeat the response categories.

Read the bolded instructions. Ensure that the participant knows only to think about how difficult the activity has been in the past month due to his/her **health**. Ask the questions exactly as written. For example, “Lifting heavy objects?” Record the participant’s response for each question for all 18 questions. In most cases, if the participant gives a “Usually did not do for other reasons” answer, you should ask him/her how much difficulty he/she would have had, had he/she done the activity. For example, if your participant says he/she did not wash dishes or dust because he/she has a house keeper, or that he/she did not have to walk up any stairs, ask him/her how much difficulty he/she would have (because of his/her health) doing those activities if he/she had to do them. If the participant gives the response of “Usually did not do for other reasons” for more than 3 questions, the questionnaire cannot be scored. Please look over the questionnaire when completed to ensure that you did not skip a question.

| Item | Usually did with no difficulty | Usually did with a little help / with little difficulty | Usually did with some/moderate help / with some difficulty | Usually did with a lot of help / a lot of difficulty | Unable to do | Usually did not do for other reasons |
| --- | --- | --- | --- | --- | --- | --- |
| 1. Getting in and out of the car | 1 | 2 | 3 | 4 | 5 | 6 |
| 2. Using the toilet including getting on and off the toilet | 1 | 2 | 3 | 4 | 5 | 6 |
| 3. Moving in and out of a chair | 1 | 2 | 3 | 4 | 5 | 6 |
| 4. Moving in and out of a bed | 1 | 2 | 3 | 4 | 5 | 6 |
| 5. Dressing yourself | 1 | 2 | 3 | 4 | 5 | 6 |
| 6. Bathing or showering | 1 | 2 | 3 | 4 | 5 | 6 |
| 7. Gripping with your hands | 1 | 2 | 3 | 4 | 5 | 6 |
| 8. Walking several blocks | 1 | 2 | 3 | 4 | 5 | 6 |
| 9. Walking one block | 1 | 2 | 3 | 4 | 5 | 6 |
| 10. Climbing several flights of stairs | 1 | 2 | 3 | 4 | 5 | 6 |
| 11. Lifting heavy objects | 1 | 2 | 3 | 4 | 5 | 6 |
| 12. Climbing one flight of stairs | 1 | 2 | 3 | 4 | 5 | 6 |
| 13. Lifting/carrying something as heavy as 10lbs | 1 | 2 | 3 | 4 | 5 | 6 |
| 14. Taking care of a family member | 1 | 2 | 3 | 4 | 5 | 6 |
| 15. Visiting with relatives or friends | 1 | 2 | 3 | 4 | 5 | 6 |
| 16. Participating in community activities | 1 | 2 | 3 | 4 | 5 | 6 |
| 17. Doing light hoursework | 1 | 2 | 3 | 4 | 5 | 6 |
| 18. Managing your money | 1 | 2 | 3 | 4 | 5 | 6 |
| 19. Using the telephone | 1 | 2 | 3 | 4 | 5 | 6 |

**4. Clinical Frailty Scale (CFS) – AV ACCESS Study Questionnaire** (see reference 29).

| **CFS Score** | **Interpretation** |
| --- | --- |
| 1 | **Very fit**: robust, energetic, well-motivated, and fit; fittest in their age group |
| 2 | **Well**: without active disease but not as fit as those in category 1 |
| 3 | **Well**: with treated comorbid disease |
| 4 | **Apparently vulnerable**: not dependent but has symptoms from comorbid disease (such as being slowed up) |
| 5 | **Mildly frail**: limited dependence on others for instrumental activities of daily living |
| 6 | **Moderately frail**: help is needed for instrumental activities of daily living and activities of daily living |
| 7 | **Severely frail**: completely dependent on others for instrumental activities of daily living and activities of daily living or terminally ill |

**5. EuroQol 5-D 3-L**

The EQ-5D-3L obtains information regarding participant’s health-related quality of life. It consists of 5 items: mobility, self-care, usual activities, pain/discomfort, and anxiety/depression (see reference 23). The participant should be informed that the questions related to the health on the day of survey. The answers to each item are categorized in 3 levels: ‘no problems’ which corresponds to a score of 0, ‘some/moderate problems’ which corresponds to a score of 2, and ‘unable/extreme problems’ which corresponds to a score of 3. All the responses generated are summed to generate an index score.

**Baseline and follow up assessment**

Under each heading, please tick the ONE box that best describes your health TODAY.

**MOBILITY**

I have no problems in walking about 

I have some problems in walking about 

I am confined to bed 

**SELF-CARE**

I have no problems with self-care 

I have some problems washing or dressing myself 

I am unable to wash or dress myself 

**USUAL ACTIVITIES** *(e.g. work, study, housework, family or leisure activities)*

I have no problems with performing my usual activities 

I have some problems with performing my usual activities 

I am unable to perform my usual activities 

**PAIN / DISCOMFORT**

I have no pain or discomfort 

I have moderate pain or discomfort 

I have extreme pain or discomfort 


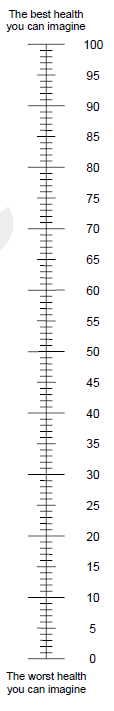

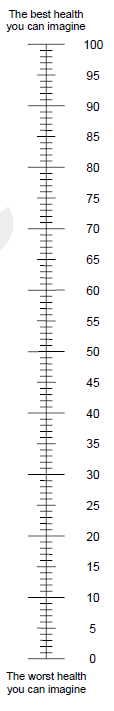

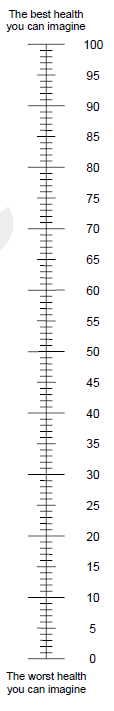


**ANXIETY / DEPRESSION**

I am not anxious or depressed 

I am moderately anxious or depressed 

I am extremely anxious or depressed 

We would like to know how good or bad your health is TODAY.

This scale is numbered from 0 to 100.

100 means the best health you can imagine.

0 means the worst h1ealth you can imagine.

Please mark an X on the scale to indicate how your health is TODAY.

Now, write the number you marked on the scale in the box below

YOUR HEALTH TODAY = ______

**6. Vascular Access Questionnaire (VAQ)** (see reference 21).

**Baseline and follow up assessment**

**Description**: This short-form questionnaire is composed of 4 vascular access-related questions.

**Important Notes**:

- When this questionnaire is administered at **baseline**, the questions refer to the participant’s satisfaction with the **catheter** they have at study enrollment. The Study Coordinator should make sure the participant understand which vascular access is addressed with the questions posed with the questionnaire.
- When this questionnaire is administered during **follow-up**, the questions refer to the participant’s satisfaction with the **index** AV access – if the participant underwent surgery for index AV access, even if the index AV access failed and did not undergo surgery for a new AV access.
- If the participant did not undergo index AV access surgery, then the questions related to the **catheter** being used for dialysis.
- If the participant had an index AV access that failed and then underwent another surgery for a new AV access intervention, then the questions should refer to the participant’s satisfaction with the **new** AV access they received.
- The Study Coordinator should make sure the participant understand which vascular access is addressed with the questions posed with the questionnaire
- The interviewer should avoid the word dialysis when posing instrument questions, as the participant may confuse the scope of the question as being related to the dialysis treatment itself rather than being related to the vascular access. Therefore, abstain from using dialysis access; instead, use either shunt (for AV access) or catheter.

**Prompts**: We would like to ask you a few questions about the catheter you have. When I am going to ask you these questions, please think of the catheter(s) you have had and the experiences you had so far with the catheter(s).

***If participant has index AV access surgery:***

I believe you had surgery to have a [shunt] placed; Is that correct?

When I am going to ask you these questions, please think of the [shunt] you had received and the experiences you had so far with the [shunt].

***If*** ***participant did not have an AV access surgery*:**
I believe you did not have surgery to have a [fistula / graft] placed; Is that correct?

When I am going to ask you these questions, please think of the catheter(s) you have had and the experiences you had so far with the catheter(s).

***If participant has a new AV access surgery:***

I believe you had another surgery to have a [shunt] placed; Is that correct?

When I am going to ask you these questions, please think of the new [shunt] you had received and the experiences you had so far with the new [shunt].

| Domain Question | Answer (circle a number on the scale that indicates your level of agreement with this statement) | | | | |
| --- | --- | --- | --- | --- | --- |
| **Physical symptoms domain:**  During the past 4 weeks, on days between dialysis sessions, I was bothered most days by pain associated with my **[shunt] [catheter]** access. | Strongly Disagree | Somewhat Disagree | No opinion | Somewhat Agree | Strongly Agree |
| **Social functioning domain:**  During the past 4 weeks my **[shunt] [catheter]** access caused me problems when bathing or showering or doing usual chores. | Strongly Disagree | Somewhat Disagree | No opinion | Somewhat Agree | Strongly Agree |
| **Complications domain:**  During the past 4 weeks my **[shunt] [catheter]** access had problems (i.e., did not work properly). | Strongly Disagree | Somewhat Disagree | No opinion | Somewhat Agree | Strongly Agree |
|  |  |  |  |  |  |
| **Overall satisfaction:**  I am satisfied with my **[shunt] [catheter**] access. | Strongly Disagree | Somewhat Disagree | No opinion | Somewhat Agree | Strongly Agree |

**7. Attitude Scale Tradeoffs**

**Baseline and follow up assessment**

**Description:** The Attitude Scale is a validated tool that elicits health outcome prioritization using items that are easily understood by and acceptable to patient. This multi-item instrument is composed of a series of statements with which participants are asked to rate the strength of their agreement (see reference 24). The questions have been adapted to the context of vascular access care.

**Prompts:** We would like to see what tradeoffs you would hypothetically be willing to consider between the intensity of vascular access care and your current and future health outcomes.

**Important Notes**:

- When the baseline interview is performed, the catheter is the type of vascular access used for hemodialysis.
- The questions posed with this instrument do not suggest that a particular type of vascular access would be better than the other. The scenarios are hypothetical to elicit participant’s points of view and potential tradeoff they would make between present and future health.
- When this instrument is administered at follow-up during the study, the participant may have either a catheter, or a shunt/AV access, or both.
- The interviewer should avoid the word dialysis when posing instrument questions, as the participant may confuse the scope of the question as being related to the dialysis treatment itself rather than being related to the vascular access. Therefore, abstain from using dialysis access; instead, use either shunt or catheter.
- The interviewer should refrain from naming the *type* of AV access the participant may have received or may be using. Instead, use the word shunt.

| **Tradeoffs between Present and Future Health** | | | | | |
| --- | --- | --- | --- | --- | --- |
| 1. The most important thing to me is living as long as I can, even if that means I have to change the type of vascular access I have now and go through more surgeries and/or more procedures with the change in vascular access. | Strongly Disagree | Disagree | Neither Agree  Nor Disagree | Agree | Strongly Agree |
| 1. I am willing to put up with more surgical interventions and more procedures for my vascular access now, if it means in the future I might be less likely to get ill. | Strongly Disagree | Disagree | Neither Agree  Nor Disagree | Agree | Strongly Agree |
| 1. *I would prefer to undergo fewer surgeries and procedures for my vascular access now, even if that means I might not live as long; or even if it means my chances of dying might be higher.* | Strongly Disagree | Disagree | Neither Agree  Nor Disagree | Agree | Strongly Agree |
| 1. *I would prefer to undergo fewer surgeries and procedures for my vascular access now, even if it means in the future I might be more likely to get ill.* | Strongly Disagree | Disagree | Neither Agree  Nor Disagree | Agree | Strongly Agree |

**8. SUPPORT Questionnaire: Goal Concordant Care Assessment** (see reference 25).

**Baseline and follow up assessment**

Note: When the baseline interview is performed, the catheter is the type of vascular access used for hemodialysis.

***First Item Description:*** This instrument elicits participant’s **healthcare goals** with respect to **vascular access options**. Participant’s answer to goals of care question is catalogued into one of the three choices. The questions have been adapted to the context of vascular access care.

**Prompts:** We are interested in the kind of vascular access you would want at this time. The question is about the relationship between the vascular access and healthcare goals.

**GOALS OF CARE QUESTION:** If you had to make a choice at this time,

- would you prefer a vascular access that focuses on extending your life as much as possible, even if it means having more surgical interventions, procedures, doctors’ visits, pain and/or discomfort with that vascular access;
- or would you want a plan of vascular access care that focuses on avoiding surgeries, procedures, doctors’ visits, pain and discomfort as much as possible, even if it means not living as long?

Participant’s answer below. Check one box.

- Extending life, even if it means having more surgeries, procedures, doctors’ visits, pain and/or discomfort with the vascular access
- Avoiding surgeries, procedures, doctors’ visits, pain and discomfort as much as possible, even if it means not living as long
- I don’t know for sure

Note: The follow up question refers to the type of AV access the patient received as part of randomization, even if it is not used for hemodialysis at the time of interview. If the patient did not undergo AV access surgery, then the question refers to the catheter. **Important Notes:**

- The interviewer should avoid the word ‘dialysis’ when posing instrument questions, as the participant may confuse the scope of the question as being related to the dialysis treatment itself rather than being related to the vascular access. Therefore, abstain from using ‘dialysis access’; instead, use either access or vascular access.
- The interviewer should refrain from naming the *type* of AV access the participant may have received or may be using (i.e., refrain from using the words ‘fistula’ or ‘graft’). Instead, use the word ‘shunt’.

***Second Item Description:***

This instrument elicits participant’s **impression** about the care he/she received for their vascular access. Participant’s answer is catalogued into one of the three choices. The questions have been adapted to the context of vascular access care.

**Prompts:** We would like to see how you feel about the vascular access care you have received so far, with regards to the outcomes, surgeries and procedures you experienced so far.

**CONCORDANCE OF CARE QUESTION:**

Based on the care you received so far concerning the vascular access(es) you have, the surgeries and/or procedures you experienced so far, which of the following best describes the focus of the access care you have been receiving?

Participant’s answer below. Check one box.

- Extending life, even if it means having more surgeries, procedures, pain and/or discomfort
- Avoiding surgeries, procedures, pain and/or discomfort, even if it means not living as long
- I don’t know, not sure

**9. Decision Regret Assessment**

**Description:** The Decision Regret Scale measures distress or remorse after a heath care decision (see reference 22). The instrument has been adapted to the context of vascular access care.

**Important Notes**:

- This instrument will **not** be administered if the participant has **not** undergone AV access surgery intervention.
- Any participant who underwent AV access surgery intervention (AVF creation or AVG placement) will receive this questionnaire, whether the AV access is used or is not used for hemodialysis at the time of assessment.
- The interviewer should avoid the word ‘dialysis’ when posing instrument questions, as the participant may confuse the scope of the question as being related to the dialysis treatment itself rather than being related to the vascular access. Therefore, abstain from using ‘dialysis access’; instead, use the word ‘shunt’.
- The interviewer should refrain from naming the *type* of AV access the participant may have received or may be using (i.e., refrain from using the words ‘fistula’ or ‘graft’). Instead, use the word ‘shunt’.
- The questions refer to the participant’s satisfaction with the **index** AV access they received -- even if the index AV access failed and did not undergo surgery for a new AV access.
- If the participant had an index AV access that failed *and* then underwent another surgery for a new AV access intervention, then the questions should refer to the participant’s satisfaction with the **new** AV access they received.

- The answers associated with scores 1 through 5 have been reverted in the last two questions relative to the first two questions, in order to maintain consistency in message description when all this instrument’s scores are summed.
- The Study Coordinator should make sure the participant understand which vascular access is addressed with the questions posed with the questionnaire.

**Prompts:** We would like to evaluate your feelings about the decision you and/or your doctors have made about having an AV access for hemodialysis. These feelings can reflect the decision itself and the experiences or medical events you went through since you had the AV access (procedures, complications, discomfort, pain with cannulation, etc). Let us know how you feel about these statements (strongly agree to strongly disagree).

| **Item** | **Scoring** | | | | |
| --- | --- | --- | --- | --- | --- |
| 1. It was the right decision to have the **[shunt] [new shunt]** surgery | Strongly Disagree | Disagree | Neither Agree Nor Disagree | Agree | Strongly Agree |
| 1. I would go for **[shunt] [catheter] [new shunt]** surgery if I had to do it over again | Strongly Disagree | Disagree | Neither Agree Nor Disagree | Agree | Strongly Agree |
| 1. I regret the decision that was made about having the **[shunt] [catheter] [new shunt]** surgery | Strongly Disagree | Disagree | Neither Agree Nor Disagree | Agree | Strongly Agree |
| 1. The **[shunt] [catheter] [new shunt]** surgery did me a lot of harm | Strongly Disagree | Disagree | Neither Agree Nor Disagree | Agree | Strongly Agree |
